# Analysis of anthropometric measurements in U-15 female weightlifters using Kinect camera and comparison with traditional methods

**DOI:** 10.1007/s11517-025-03373-3

**Published:** 2025-05-16

**Authors:** Bülent Işik, Kenan Erdaği, Serkan Örücü, Usame Ömer Osmanoğlu, Erkan Özbay

**Affiliations:** 1 Departments of Physiology, Medical School, University of Karamanoğlu Mehmetbey, 70200 Karaman, Turkey; 2 Departments of Physical Education and Sports, Ahmet Keleşoğlu Faculty of Education, Necmettin Erbakan University, 42090 Konya, Turkey; 3 Vocational School of Technical Sciences, Computer Technologies Program, Karamanoğlu Mehmetbey University, 70200 Karaman, Turkey; 4 Departments of Biostatistics, Medical School, University of Karamanoğlu Mehmetbey, 70200 Karaman, Turkey; 5 Karamanoğlu Mehmetbey University Vocational School of Health, 70200 Karaman, Turkey

**Keywords:** Microsoft Kinect, Anthropometric measurement, Olympic style weightlifting, U-15 age category, Adolescent female weightlifters

## Abstract

Anthropometric measurements are important in a wide range of areas, from athlete selection to performance analysis and medical exercise applications. With its portable and cost-effective structure, Microsoft Kinect offers significant advantages in measuring human movements and provides valuable data in the fields of sports science and medicine. The aim of this study is to compare certain anthropometric measurements of adolescent female weightlifters in the U-15 age category with Kinect V2 and traditional methods. Twelve athletes who won medals in international weightlifting championships were included in the study. Anthropometric evaluations of each athlete were performed using Kinect V2 and the traditional method. Differences between measurements were analyzed with Bland–Altman plots and Pearson correlation coefficients. Kinect was found to exhibit less variability and higher coherency in measurements of humerus, forearm, trochanter-tibiale laterale, tibial lengths, and shoulder width. It has been observed that the traditional method provides more coherence results in hand length measurements. Kinect was found to exhibit less variability and higher coherency in measurements of forearm, trochanter-tibiale laterale, tibial lengths, and shoulder width, indicating its potential as a reliable tool for these parameters. Notably, Kinect demonstrated high reliability for tibial length (ICC 0.918) and moderate reliability for trochanter-tibiale laterale (ICC 0.737), showing its effectiveness in these measurements. Additionally, Kinect exhibited lower variation and higher coherency in most measurements compared to manual methods, supporting its consistency and repeatability in anthropometric assessments. These results indicate that Kinect may be a suitable tool for some measurements but that traditional methods may be preferable for hand length measurements. These findings suggest that Kinect can be used effectively for certain anthropometric measurements in sports and medical science.

**Graphical Abstract:**

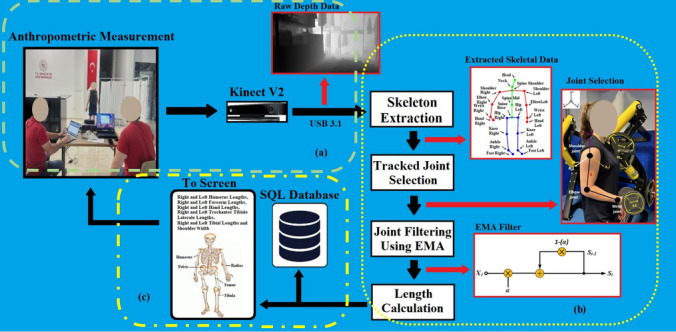

## Introduction

Anthropometry, related to body dimensions, includes measurements such as length, width, circumference, and skinfold thickness [1]. Anthropometric characteristics encompass variable attributes that are influenced by individual physiological and structural factors such as gender, sports activity, nutrition, genetic factors, and general body health [2–4]. The conformity of anthropometric characteristics is of great importance not only in the selection of athletes but also in the employment of people in various sectors. In addition, the role of anthropometric characters is indispensable in talent detection and the display and development of sports performance [5–7]. Anthropometric measurements are widely preferred both for determining simple parameters related to body form and for indirect estimation of body composition using regression models [8]. Anthropometric analyses are used in a wide range of fields such as scientific research, sports studies, medicine, rehabilitation, biomechanics, and clothing industry [9–13]. In measurements applied in this branch of science based on anthropometric measurements, the implementation of an appropriate measurement procedure sometimes necessitates the use of special measurement instruments (e.g., anthropometer, measuring tapes, and calipers), which can be costly. In addition, the measurement process is often complex, uncomfortable for the individual being measured, and time-consuming; trained personnel are required to carry out this process effectively [1, 13–15]. Considering the above, it becomes logical to look for different measurement methods that can support or partially replace existing techniques or tools. As an alternative to classical anthropometry, dual energy X-ray absorptiometry (DXA), computed tomography (CT) and magnetic resonance imaging (MRI), which are imaging methods widely used in medicine, stand out [14, 16, 17]. However, it has been stated that these techniques require time and cost and can expose the body to invasive X-ray radiation. In recent years, it has been reported that 3D scanning models demonstrate a superior performance compared to standard anthropometric measurement methods in estimating body composition [17–19]. When the literature reviews are examined, nowadays, the limitations mentioned above are overcome by the use of anthropometric measurement systems working with computer-based techniques such as three-dimensional body scanners. Body scanners have proven their effectiveness as an efficient and versatile piece of equipment that requires less time and is less invasive than manual measurement [20, 21]. In the group of systems and tools used to estimate anthropometric parameters of the human body, the Kinect sensor has found a wide range of applications. With its low price and features such as being equipped with an RGB camera, an infrared camera and an infrared transmitter, this device has become an important element of scientific research worldwide [20, 22–24]. Kinect sensors have been used successfully in many fields such as medicine [25], medical exercises [26], anthropometric measurement [14, 22, 23, 27, 28] and sports [29, 30]. In a study in which a system was developed to estimate human anthropometric parameters based on three-dimensional scanning of the whole body using the low-cost Kinect v2 sensor, it has been stated that the developed system creates a 3D human model based on the data obtained from the depth sensor, and then by segmenting this model, it can measure the anthropometric parameter values representing the human structure at a satisfactory level, and this method can be used effectively in anthropometric measurements [31]. In another study using the Kinect camera, it was stated that a graphical user interface that allows automatic calculation of height and weight in the human body was successfully developed [31]. In the research where human body measurements were performed using the Kinect sensor and this technology was integrated into the fashion industry, it was explained that especially the length anthropometric measurement values in the human body can be measured with very low error rate percentages [22]. In the study comparing anthropometric data collected using a manual anthropometric measurement method and a Kinect-based body imaging system, it was reported that there was no significant difference between neck circumference, calf circumference and chest length measurements [23]. It has been stated that all extremity lengths were recorded during medical exercises using the Kinect system. It has been explained that the data obtained can be used to guide and control home-based medical exercises [26]. The use of Kinect motion capture techniques not only provides technical guidance in physical education and sports training, but also plays an active role in sports activities. The application of motion capture technology in physical education teaching and training has the potential to effectively increase the performance of competitive athletes through the design of Kinect-based sports training systems. This technology is considered an important tool to improve the performance of athletes and increase the quality of sports training [30]. It has been reported that identifying and correcting incorrect postures of swimmers significantly increases the quality of daily swimming training athletes. It has been stated that the reason why coaches cannot detect and correct poor postures of athletes in real time is due to inadequate detection and correction. The authors successfully calculated the position information of swimmers in motion using Kinect technology and stated that this information is of critical importance in detecting, recognizing, and correcting improper postures of swimming athletes [29].

Kinect and computer-based systems increase the accuracy of anthropometric measurements, making the measurement processes faster, more practical and reliable. The high reliability offered by these systems can provide significant advantages for both researchers and athletes in the field of sports science. These technologies can play a critical role in the evaluation of body composition and physical characteristics of adolescent female athletes. In addition, the possibility of reaching adolescent female weightlifters who regularly do weightlifting at an athletic level and have achieved international success is quite limited, as weightlifting is a difficult sport for the female gender by nature. For this reason, the current study was intended to be conducted on accessible adolescent female elite female weightlifting athletes. The accuracy of Kinect V2 in anthropometric measurements may be influenced by specific physiological characteristics of the population being studied. Elite female weightlifters, due to their high muscle density and intensive training regimens, present a unique body composition that may affect measurement precision. Thus, including this group allows for a focused evaluation of Kinect V2’s applicability in high-performance athletes while also providing insights into its broader use in sports science. This emphasis on monitoring physical characteristics, besides physiological status monitoring, is extremely important in optimizing training effectiveness among adolescent female weightlifters. In this way, performances of athletes can be analyzed in more detail and precisely, and individual training programs can be created more effectively. In addition, these technologies can help adolescent athletes to keep their health and performance at the highest level by contributing to the early detection and prevention of possible physical problems. Based on these considerations, the aim of this study is to analyze some anthropometric values of adolescent female weightlifting athletes in the Under-15 (U 15) age category using traditional methods and Kinect V2. Additionally, it is to identify the measurement differences of these two different methods. In this way, we aim to evaluate the accuracy, practicality, and reliability of these technologies in the field of sports and medicine science.

## Materials and methods

### Participants and data collection

Our research covers 12 volunteer adolescent female weightlifting athletes who do not have health problems and who prepare for international championships by training twice a day, 6 days a week, and who won a medal in the European U-15 Weightlifting Championship held in Thessaloniki, Greece (15–23 June 2024). Demographic, anthropometric, and weightlifting performances of the athletes who participated in anthropometric measurement analyses performed with Kinect V2-based and manual (traditional) methods are presented in Table 1. Athletes were those who trained at least 6 days per week for double training and were free of orthopedic injuries that would preclude weightlifting training within the past 6 months. Athletes with a history of serious trauma in their lower and upper extremities, or any structural or systemic disease causing joint or movement limitations were not included in the study. All inclusion and exclusion criteria were evaluated by an attending experienced in sports practice, through examination and questioning techniques. Prior to participation, informed consent was obtained from each participant and their parent/legal guardian. Written informed consent was obtained from all individuals whose images appear in this study. This study was performed in accordance with the principles of the 2013 Helsinki Declaration. All procedures were approved and endorsed by the institutional review board prior to testing (Karamanoğlu Mehmetbey University Medicine Faculty Scientific Ethics Committee; approval number; 08–2024/11, Karamanoğlu Mehmetbey University Scientific Research Projects Coordination Unit, Project No: 14-M-24). Before starting the research, a brief explanation about the study was given to the participants. Anthropometric measurements were performed according to techniques described by Norton [32]. In order to increase the reliability of the measurements, each participant was measured three times with traditional manual methods and three times using the Microsoft Kinect V2 device, and the average of these three values was used for subsequent data processing stages. Participants wore their own underwear and T-shirts throughout the measurement process. It is of great importance that underwear and t-shirts fit the body without being tight, so as not to affect body shape, and during measurements, athletes’ clothing was determined in accordance with the measurement techniques. For manual measurements, participants were asked to maintain stabilization in exactly the same posture positions and hold their breath in order to avoid differences in results due to different postures. Before starting the manual data collection process, the body region must be determined. Before starting the research, the regions to be measured were determined with a marker pen.

**Table 1:** Demographic, anthropometric, and weightlifting performance values of female weightlifters

	Parameters	Mean	Standard deviation	Median	Q1–Q3
	Age (year)	14.67	0.49	15.00	14.00–15.00
	Height (cm)	159.58	4.96	157.50	156.25–164.50
	Body weight (kg)	56.00	13.21	57.00	45.00–62.70
	BMI (kg/m^2^)	21.82	4.16	21.45	18.48–25.03
	Training age (year)	4.00	1.28	3.50	3.00–5.50
	Max snatch (kg)	68.17	8.53	71.50	59.25–75.25
	Max clean and jerk (kg)	85.08	9.14	86.50	78.25–92.75

*BMI*, body mass index (kg/m^2^), max snatch, maximum snatch weightlifting performance (kg), Max clean and jerk, maximum clean and jerk weightlifting performance (kg), Q: quartile

### Sample size calculation

The calculation of sample size was based on the study by Krzeszowski et al., 2023 [31]. The automatic direct method available in G*Power software 3.1.9.7 version was used, with a medium effect size of 1.06, the significance level of *α* = 0.05, power = 0.80. The sample size obtained was calculated to be at least 10 weightlifters. In addition, 20% participants were added to compensate for possible problem. Thus, the sample size to be studied was determined as 12 weightlifters. In addition, according to the results of the study conducted with 12 participants’, the effect size (d) was calculated as 1.029 and the power as 89.9% according to the difference between two dependent groups at the significance level of *α* = 0.05.

### Kinect V2 features and system design

Released in 2014, and with main features outlined in Table 2, Kinect V2 is a multi-component sensor developed by Microsoft that provides depth perception, gesture recognition and human–computer interaction.

**Table 2:** Kinect V2 features

Features	Details
Colour camera	1920 × 1080 resolution, 30 fps (frames per second)
Audio acquisition	Four microphone arrays, 16-kHz sampling rate
Depth sensor	512 × 424 resolution, 30 fps (frames per second)
Minimum operating distance	0.5 m
Maximum operating distance	4.5 m
Field of view	70° horizontal, 60° vertical

Kinect V2 is equipped with an RGB camera that has a resolution of 1920 × 1080 pixels, which is capable of recording color images at 30 frames per second (fps) and synchronizing them with depth data. In addition, the array of four omnidirectional microphones included with the device can determine the location of the sound source and collect clear audio data with noise filtering. The operational range of Kinect V2 extends from 0.5 to 4.5 m, while its horizontal viewing angle is 70° and its vertical viewing angle is 60°. Kinect V2’s depth sensor consists of an infrared CMOS camera (512 × 424 pixels and a sampling rate of 30 fps) working in conjunction with three IR transmitters [33, 34], (see Table 2).

With TOF (time-of-flight) technology, each pixel is provided separately and a depth map is created [35, 36]. Next, the pixel positions in the resulting depth map are converted to real-world coordinate data as shown in Eqs. 1 and 2.

1 $${X}_{real}=\frac{\left({X}_{pixel}-{C}_{x}\right).{Z}_{real}}{{f}_{x}}$$

2 $${Y}_{real}=\frac{\left({Y}_{pixel}-{C}_{y}\right).{Z}_{real}}{{f}_{y}}$$

**Fig. 1:**
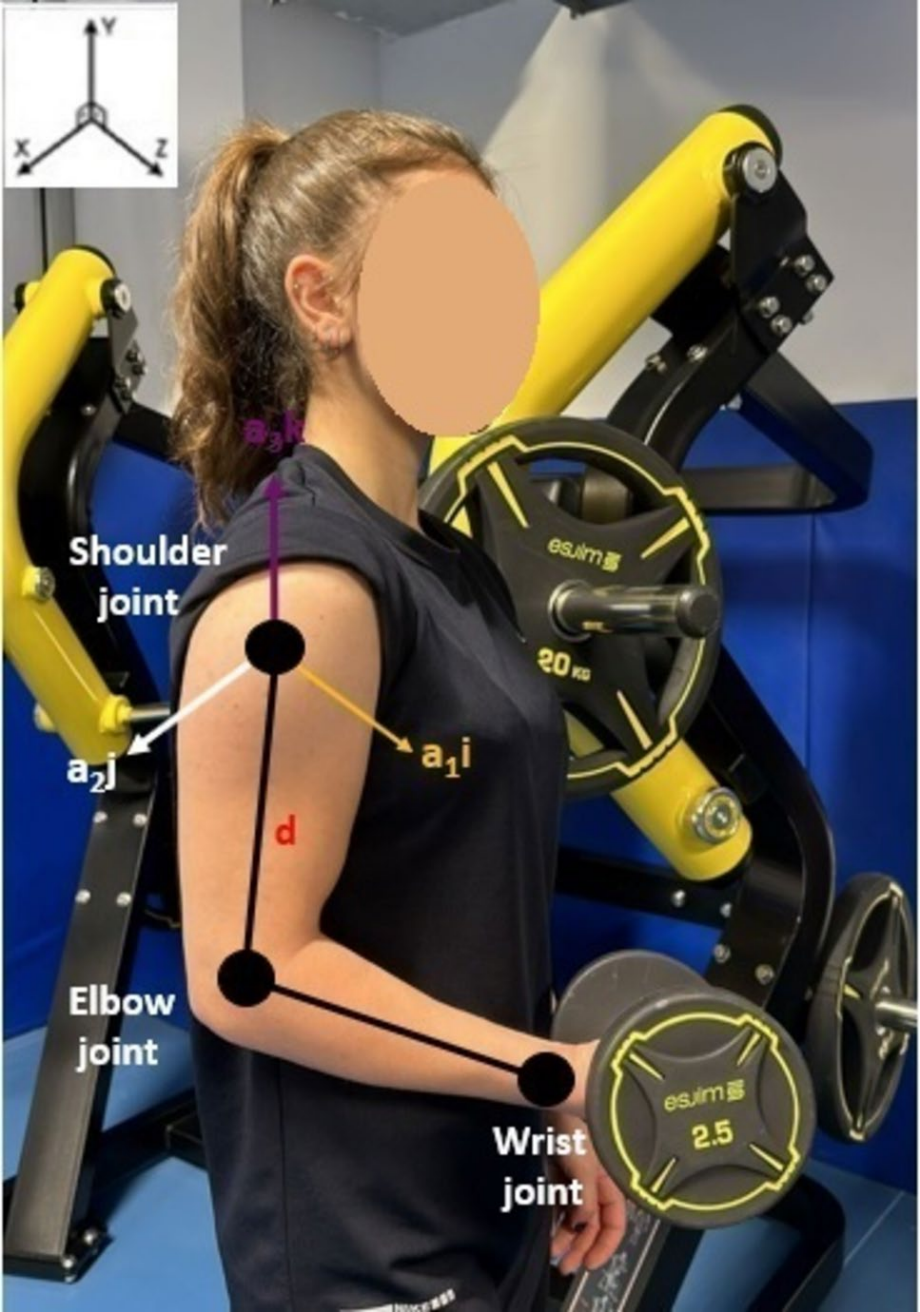
Demonstrations of distance between joints vectors (shoulder-elbow, elbow-wrist joints)

In *Y* and *Z*, *X*_pixel_ and *Y*_pixel_ represent the position of the pixel in the depth map, *X*_real_, *Y*_real_, and *Z*_real_ represent the real-world coordinates, *cx* and *cy* represent the coordinates of the camera optical center, and finally *fx* and *fy* represent the camera focal lengths. Thus, using perspective geometry and camera features, 3D coordinates of any point are obtained as shown in Fig. 1 [37].

The depth of any pixel in each depth data frame obtained from Kinect V2 can be converted to three-dimensional coordinates according to the triangulation principle as shown in Eq. 3. In this way, it allows the 3D coordinates of any joint to be calculated with the information obtained from the sensor.

In Eq. 3, “*x*_*p*_” represents the horizontal coordinate of a pixel in the depth image, “*y*_*p*_” represents the vertical coordinate of a pixel in the depth image, “*z*_*p*_” represents the depth value of the pixel in these coordinates, “*p*_*h*_” represents the total number of pixels in the horizontal direction, “*p*_*v*_” represents the total number of pixels in the vertical direction, “*θ*_*h*_” represents the horizontal viewing angle of the IR camera and “*θ*_*v*_” represents the vertical viewing angle of the IR camera.

3 $$\begin{array}{l}X=\frac{\left({X}_{ p}-{P}_{h}/2\right)\text{tan}\left({~}^{{\theta }_{h}}\!\left/ \!{~}_{2}\right.\right)}{{~}^{{P}_{h}}\!\left/ \!{~}_{2}\right.}{Z}_{p},\\ Y=\frac{\left({P}_{v}/2-{Y}_{p}\right)\text{tan}\left({~}^{{\theta }_{h}}\!\left/ \!{~}_{2}\right.\right)}{{~}^{{P}_{v}}\!\left/ \!{~}_{2}\right.}{Z}_{p},\\ Z={Z}_{p}\end{array}$$

Studies have shown that there are distortions called noise in the depth data obtained from Kinect V2 [38, 39]. It is reported that these noises may occur due to various sources such as sensor limitations, environmental conditions, and object properties [37, 40]. Therefore, in order to obtain more accurate results, it is important to filter out these noises, smooth the data, and increase the reliability of the measurements [41, 42]. For this reason, the Exponential Moving Average (EMA) filter, which was also preferred in Chavan’s and Wang’s [43, 44] researches, was used in our study because of its fast, reliable and sensitive characteristics. The EMA Filter is an infinite impulse response filter that uses exponentially decaying weighting [45]. With this feature, the weighting used for each old data decreases exponentially and never reaches zero [46]. Thus, the EMA filter provides some degree of smoothing on old data while still being sensitive to recent changes. When applying the EMA filter to 3D joint coordinates in our system, we treated each joint point independently as done in Örücü and Selek’s study [47]. Then, we applied the filtering process separately to the *X*, *Y*, and *Z* components of the joint coordinates specified in Eq. 3, as expressed in Eq. 4.

4 $$\begin{array}{l}{S}_{x,t}=\alpha .{X}_{t}+\left(1-\alpha \right).{S}_{x,t-1},\\ {S}_{y, t}=\alpha .{Y}_{t}+\left(1-\alpha \right).{S}_{y,t-1,}\\ {S}_{z,t}=\alpha .{Z}_{t}+\left(1-\alpha \right).{S}_{z,t-1}\end{array}$$

In Eq. 4, “*S*_*x,t*_, *S*_*y,t*_, and *S*_*z,t*_” represent the corrected values for *X*, *Y*, and *Z* coordinates at time *t*, respectively; *α* is the smoothing factor, “*X*_*t*_, *Y*_*t*_, and *Z*_*t*_,” represent the measured values of joint coordinates at time *t*, and “*S*_*x*,*t*−1_, *S*_*y*,−1_, and *S*_*z*,*t*−1_,” represent the corrected values at the previous time step *t-*1.

Following the reduction of noise in the coordinates of the joints in *R*^3^ using the EMA filter, the joints tracked in the study were selected. After this process, vectors were created for these tracked joints. In this process, the nonzero A vector formed between the wrist and elbow, and the nonzero B vector formed between the elbow and shoulder are defined in the *R*^3^ as *A* = *a*_1_*i* + *a*_2_*j* + *a*_*3*_*k and B* = *b*_1_*i* + *b*_2_*j* + *b*_3_*k*. These vectors were presented in Eqs. 5 and 6, with $\left\|\overrightarrow A\right\|$ denoting the length of vector A and $\Arrowvert\overrightarrow B\Arrowvert$ denoting the length of vector B.

5 $$\Vert \overrightarrow{A}\Vert =\sqrt{{\left({Shoulder}_{Left}.x\right)}^{2}+{\left({Shoulder}_{Left}.y-{Elbow}_{left}.y\right)}^{2}+{\left({Shoulder}_{Left}.z-{Elbow}_{Left}.z\right)}^{2}}$$

6 $$\Vert \overrightarrow{B}\Vert =\sqrt{{\left({Elbow}_{Left}.x-{Wrist}_{Left}.x\right)}^{2}+{\left({Elbow}_{Left}.y-{Wrist}_{Left}.y\right)}^{2}+{\left({Elbow}_{Left}.z-{Wrist}_{Left}.z\right)}^{2}}$$

Subsequent to the process outlined above, the distance between the joints, “*d*”, is calculated using the data obtained for each joint coordinate using 5 and 6, as demonstrated in Eq. 7.

7 $$d=\sqrt{\sum_{i=1}^{N}{\left({A}_{i}-{B}_{i}\right)}^{2}}$$

The last process in the system is to present the distances between the joints to the users through the interface and save them in the database, as explained above.

### Experimental study

**Fig. 2:**
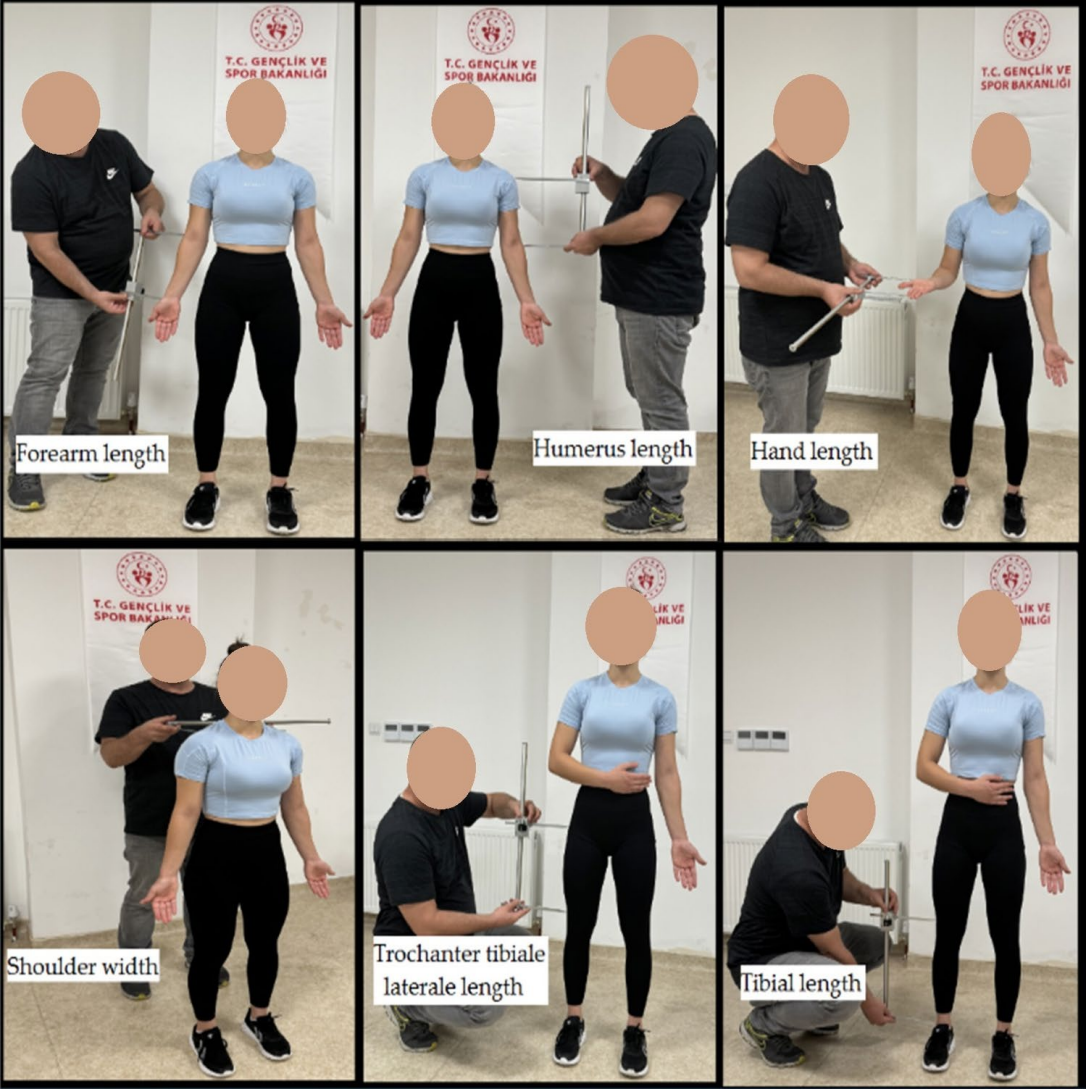
Manual anthropometric measurements (forearm, humerus, hand, trochanter tibiale laterale, tibial lengths and shoulder width)

Our experimental study was performed in two stages. In the first stage, the Harpender digital caliper (Holtain Limited), a manual anthropometry measurement set, was used in the traditional measurement method for measuring the lower extremity, upper extremity length (right-left) and shoulder width of weightlifters (Fig. 2). Manual anthropometric measurements were performed by an experienced medical doctor by measuring three times from both sides in a relaxed standing position and recording the average value of the measurements in centimeter (cm). The body weights of the athletes were measured in the morning on an empty stomach, with bare feet and light clothing, using a Tanita (Tanita-MC 580, Japan) bioimpedance measurement system [48]. Body mass index (BMI) was calculated by dividing body weight by height squared. The athletes’ height measurements were recorded barefoot and using a Seca (213 portable mechanic, Germany) height scale [32]. The data on the maximal weights lifted by the athletes in the snatch and clean and jerk techniques in the U-15 European Championship were obtained from the official website of the European Weightlifting Federation [49].

Humerus length (acromiale-radiale) was measured by identifying the acromiale and radiale anatomical landmarks and using a caliper to determine the linear distance between these points. Forearm length was measured as the linear distance between the radiale and stylion anatomical landmarks using a caliper, with the athlete in a relaxed standing position. Hand length was measured with the elbow flexed, the forearm in supination, and fingers extended, determining the shortest distance between the midstylion and dactylion points. Trochanter-tibiale laterale length was measured as the linear distance between the trochanter and tibiale laterale landmarks using a caliper, with the athlete in a standing position. Tibial length was measured vertically from the lateral tibial point to the distal lateral malleolus with the athlete standing upright. Shoulder width was measured between the outermost lateral points of the acromion processes using a caliper placed at a 30° upward angle, with the athlete standing comfortably and arms relaxed at sides. Iliospinale-tibiale laterale length was measured from the iliospinale to the tibiale laterale landmarks using a caliper, with the athlete standing upright and arms relaxed. Crista iliaca-tibiale laterale length was measured using a caliper from the upper edge of the crista iliaca to the tibiale laterale landmark, with the athlete standing relaxed [50, 51] (see Fig. 2).

**Fig. 3:**
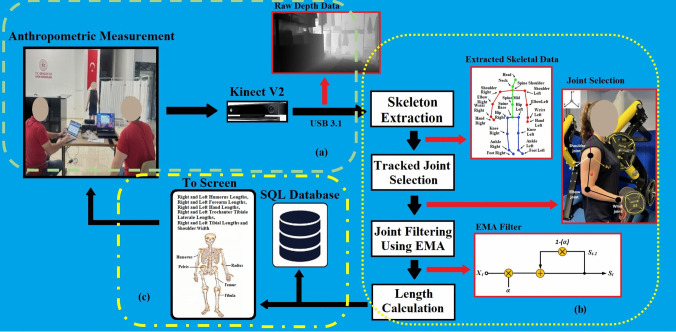
Working principle of the system developed using Kinect V2

In the second step, the data regarding the lower extremity and upper extremity lengths (right side-left side) and shoulder width of the weightlifting athletes were measured and recorded under the supervision of an expert weightlifting coach, through the system developed using Kinect V2, as shown in Fig. 3.

In this system, Kinect V2 was placed 1.5 m above the ground, and the athletes were positioned 2.5 m away from Kinect V2, as shown in Fig. 3a and reported in other studies in the literature [27]. Pure depth data obtained from Kinect V2 was transferred from the USB 3.1 port of a computer with Intel(R) Core (TM) i7, 16 GB RAM and 8 GB graphics card, as shown in Fig. 3b. First, EMA filter was applied to the transferred depth-only data due to the noise they contained, and then skeleton extraction was performed from the filtered data. 3D joint coordinates were obtained from the data obtained after skeletal extraction, and these data were used to calculate the limb lengths of weightlifting athletes. Finally, the obtained anthropometric data were recorded in the SQL database and displayed on the screen for users to follow simultaneously, as shown in Fig. 3c.

### Statistical analysis

IBM Statistical Package for Social Sciences 25.0 (SPSS, Chicago, IL) program was used for statistical analyses. For descriptive statistics of all variables, mean, standard deviation, median, 1 st quartile and 3rd quartile values were calculated. Test–retest reliability was assessed using Intraclass Correlation Coefficient (ICC) values, and the precision and repeatability of measurements were evaluated using coefficient of variance (CV) and coefficient of repeatability (CR). The Mann–Whitney *U* test was used to evaluate the significance of differences. The concordance of the measurement methods was evaluated with the Python 3.7.9 software program using Bland–Altman charts. Pearson correlation coefficients were calculated for relationships between measurements. The repeatability, variance, and inter-rater agreement coefficients of the measurements were calculated. Interpretation of the resulting ICC values was based on the categories for less than 0.5 are indicative of poor reliability; values between 0.5 and 0.75 indicate moderate reliability, values between 0.75 and 0.9 indicate good reliability, and values greater than 0.90 indicate excellent reliability [52]. The level of statistical significance was determined as *p* < 0.05.

## Results

### Main results

The values of the demographic, anthropometric, and weightlifting performances of the U-15 female weightlifters participating in the research are presented in Table 1.

**Fig. 4:**
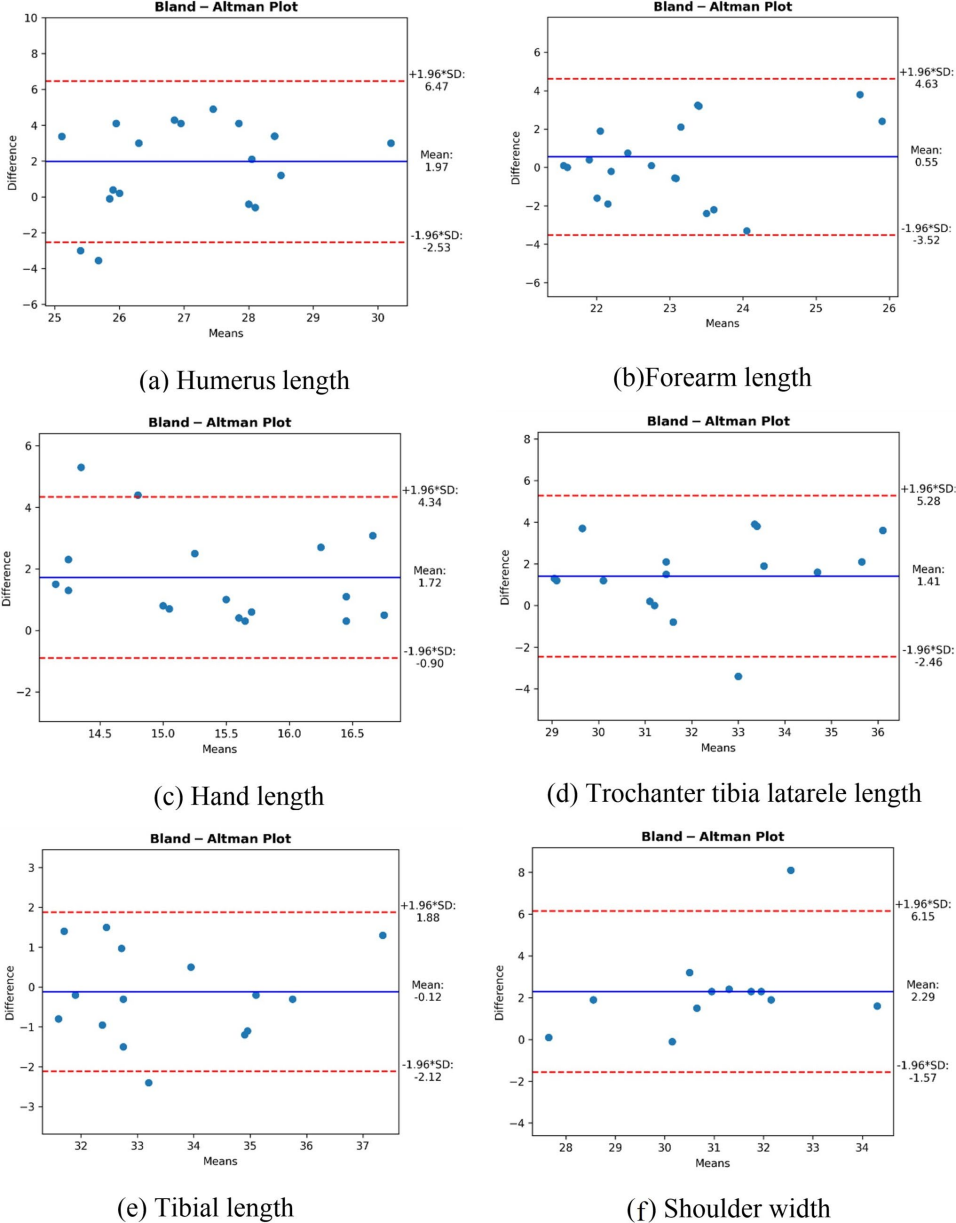
Bland–Altman plots demonstrating the agreement between Kinect V2 and manually measured for the analysis of kinematic variables (forearm, humerus, hand, trochanter tibiale laterale, tibial lengths, and shoulder width)

In humerus length measurements, Mann–Whitney *U* test results show a statistically significant difference between Kinect and manual measurements (*p* < 0.001, Table 3). This shows that the measurement results differ significantly between the two methods. As observed in Table 4, Kinect measurements show lower variation (CV 6.34% vs. 7.5%) and greater coherency (CR 4.57 vs. 5.82) compared to manual measurements. This suggests that Kinect offers more coherence and repeatable results. A low ICC (0.263) value indicates low concordance and reliability between the two methods. This suggests that manual measurements may be more reliable in terms of accuracy. Bland–Altman analysis average measurement difference is + 1.97 cm, and it is observed that the 95% confidence interval varies between − 2.53 and + 6.47 cm. This wide confidence interval indicates that the differences between the two methods can vary significantly and that there may be discrepancy between Kinect and manual measurements (Fig. 4). In general, if we make an evaluation, while Kinect provides an advantage in terms of coherency and repeatability in humerus length measurements, Kinect provides a weaker advantage in terms of measurement reliability. On the other hand, the significant difference in measurements and the low ICC value suggest that manual measurements are more reliable in terms of accuracy. However, in situations where coherency and repeatability are important in our research, Kinect may be appropriate. We think that the reason for the low concordance of Kinect with manual measurement may be due to the difference in the standard of manual measurement management of the anatomical land markers used in humerus measurement.

**Table 3:** Comparison of anthropometric variables measured using Microsoft Kinect and manual methods

Length and width measurements	Manually measured value (cm)				Microsoft Kinect measured value (cm)				*p* value
	**Mean**	**SD**	**Median**	**Q1–Q3**	**Mean**	**SD**	**Median**	**Q1–Q3**	
Humerus length (right/left)	28.00	2.10	27.90	26.28**–**29.70	26.03	1.65	25.90	24.80**–**27.34	**0.001**
Forearm length (right/left)	23.51	2.00	22.80	22.15**–**24.80	22.96	1.35	22.75	21.77**–**24.45	0.426
Hand length (right/left)	16.45	0.96	16.55	15.50**–**17.00	14.73	1.30	14.95	13.70**–**15.80	**0.001**
Trochanter-Tibiale laterale length (right/left)	33.15	2.63	31.85	31.20**–**35.45	31.75	2.33	31.75	29.73–34.20	0.099
Tibial length (right/left)	33.52	1.94	32.90	31.93–34.85	33.64	1.80	33.20	32.00–35.43	0.598
Shoulder width	32.18	2.39	32.30	30.43–33.10	29.89	1.64	30.00	28.60–30.75	**0.014**

*SD*, standard deviation; *Q*, quartile

**Table 4:** Correlation and Bland–Altman analysis results of anthropometric measurements in U-15 adolescent female weightlifters: comparison of manual and Microsoft Kinect Methods

Parameter	Manually measured value (cm)				Microsoft Kinect measured value (cm)				ICC	***r***	Bland–Altman plot	
	**Mean**	**SD**	**CV**	**CR**	**Mean**	**SD**	**CV**	**CR**			**Bias**	**95% CI (%)**
Humerus length (right/left)	28.00	2.10	7.5	5.82	26.03	1.65	6.34	4.57	0.263	0.235	+ 1.97	− 2.53 to + 6.47
Forearm length (right/left)	23.51	2.00	8.51	5.54	22.96	1.35	5.88	3.74	0.372	0.246	+ 0.55	− 3.52 to + 4.63
Hand length (right/left)	16.45	0.96	5.84	2.66	14.73	1.30	8.83	3.6	0.240	0.300	+ 1.72	− 1.57 to + 5.28
Trochanter-Tibiale laterale length (right/left)	33.15	2.63	7.93	7.29	31.75	2.33	7.34	6.46	0.737	0.675	+ 1.41	− 2.12 to + 5.28
Tibial length (right/left)	33.52	1.94	5.79	5.38	33.64	1.80	5.35	4.99	0.918	0.847	− 0.12	− 2.12 to + 1.88
Shoulder width	32.18	2.39	7.43	6.62	29.89	1.64	5.49	4.55	0.477	0.531	+ 2.29	− 1.57 to + 6.15

*SD*, standard deviation; *CV*, coefficient of variance; *CR*, coefficient of repeatability; *ICC*, intraclass correlation coefficient; *CI*, confidence interval

The Mann–Whitney *U* test results for Forearm Length measurements show that there is no statistically significant difference between Kinect and manual measurements (*p* > 0.05; Table 3). This indicates that both methods provide similar accuracy. It is observed that Kinect measurements show lower variation (CV 5.88% vs. 8.51%) and higher coherency (CR 3.74 vs. 5.54) compared to manual measurements. This suggests that Kinect offers more coherence and repeatable results. The low ICC value indicates limited concordance between the two methods (see Table 4). The data we obtained suggest that there may be some differences in the repeatability of the measurements. The mean measurement difference in Bland–Altman Analysis is + 0.55 cm, and the 95% confidence interval is observed to vary between − 3.52 and + 4.63 cm. This wide confidence interval indicates that the differences between the two methods may vary somewhat between measurements (see Fig. 4). In general, if we make an evaluation, there is no significant difference between Kinect and manual methods in forearm length measurements, indicating that both methods offer similar accuracy. However, Kinect’s lower variability and higher coherency make it a preferred method, especially when coherency and repeatability are important. Although the low ICC value indicates that there may be some differences between measurements, the coherency advantage offered by Kinect may make this method more attractive in terms of repeatability.

The Mann–Whitney *U* test results for hand length measurements show a statistically significant difference between Kinect and manual measurements (*p* < 0.001, Table 3). This result shows that the measurement results differ significantly between the two methods. It is observed that manual measurements show lower variation (CV 5.84% vs. 8.83%) and higher coherency (CR 2.66 vs. 3.6) compared to Kinect measurements. Therefore, it can be said that the manual method provides more reliable and coherence results. A low ICC value (0.240) indicates low concordance and reliability between the two methods (see Table 4). Therefore, the results obtained indicate that manual measurements can be found reliable in terms of accuracy. Bland–Altman Analysis average measurement difference is + 1.72 cm, and it is observed that the 95% confidence interval varies between − 1.57 and + 5.28 cm. This shows that the differences between the two methods can vary significantly and there can be discrepancy between Kinect and manual measurements (see Fig. 4). In general, if we make an evaluation, there is a significant difference between Kinect and manual measurements in hand length measurements, and the low ICC value suggests that manual measurements are more reliable in terms of accuracy and coherency. Manual methods may be a more appropriate choice for hand length measurements because they offer less variability and higher coherency. We think that the reason for the low concordance of Kinect with manual measurement may be due to the standard difference in manual measurement management of the anatomical land markers used in hand length measurement.

The Mann–Whitney *U* test results for trochanter-tibia laterale length measurements show that there is no statistically significant difference between Kinect and manual measurements (*p* > 0.05, Table 3). This indicates that both methods provide similar accuracy. The findings show that Kinect measurements have lower variation (CV 7.34% vs. 7.93%) and higher coherency (CR 6.46 vs. 7.29) compared to manual measurements. The results show that Kinect offers more coherence and repeatable results. The moderate ICC value (0.737) indicates good concordance and reliability between the two methods (see Table 4). This suggests that the measurements are generally reliable. Bland–Altman Analysis average measurement difference is + 1.41 cm, and the 95% confidence interval varies between − 2.12 and + 5.28 cm. This is an indication that the differences between the two methods are generally within an acceptable range (see Fig. 4). As a general evaluation, there is no significant difference between Kinect and manual methods in trochanter-tibiale laterale length measurements, indicating that both methods offer similar accuracy. However, Kinect’s lower variability and higher coherency make it a preferred method, especially when coherency and repeatability are important. The medium ICC value indicates that the Kinect method is also reliable, but the coherency advantage offered by Kinect makes this method preferable. The Mann–Whitney *U* test results for tibial length measurements show that there is no statistically significant difference between Kinect and manual measurements (*p* > 0.05, see Table 3). This indicates that both methods offer similar accuracy. Kinect measurements have been observed to exhibit lower variation (CV 5.35% vs. 5.79%) and higher coherency (CR: 4.99 vs. 5.38) compared to manual measurements. These findings suggest that Kinect offers more coherence and repeatable results. The high ICC value (0.918) indicates excellent concordance and reliability between the two methods. These results indicate that both Kinect and manual measurements are reliable in measuring tibial length (see Table 4). Bland–Altman Analysis showed that the average measurement difference was − 0.12 cm, and the 95% confidence interval varied between − 2.12 and + 1.88 cm. These data indicate that the differences between the two methods are relatively small, and the measurements are generally in concordance (see Fig. 4). In general, if we make an evaluation, there is no significant difference between Kinect and manual methods in tibial length measurements, indicating that both methods offer similar accuracy. However, Kinect’s lower variability and higher coherency make it a preferred method, especially when coherency and repeatability are important. In addition, the high ICC value emphasizes that Kinect provides high reliability and high concordance in its measurements and that this method is an effective tool for tibial length measurements.

The Mann–Whitney *U* test results for shoulder width measurements show a statistically significant difference between Kinect and manual measurements (*p* < 0.001, Table 3). This indicates that the measurement results differ significantly between the two methods. Kinect measurements show lower variation (CV 5.49% vs. 7.43%) and higher coherency (CR 4.55 vs. 6.62) compared to manual measurements. These results suggest that Kinect provides more coherence and repeatable data. A moderate ICC value (0.477) indicates poor concordance and reliability between the two methods. Therefore, this leads to the thought that there may be some differences in the repeatability of measurements (see Table 4). Bland–Altman Analysis mean measurement difference is + 2.29 cm, and the 95% confidence interval varies between −1.57 and + 6.15 cm. This suggests that the differences between the two methods are relatively wide-ranging and that there may be some discrepancies between Kinect and manual measurements (see Fig. 4). In general, if we make an evaluation, there is a significant difference between Kinect and manual methods in shoulder width measurements, suggesting that manual measurements can be reliable in terms of accuracy. However, Kinect's lower variability and higher coherency support its preference, especially in cases where coherency and repeatability are important. Although the moderate ICC value indicates that there may be some variation between measurements, the coherency advantage offered by Kinect may make this method more attractive in terms of repeatability. We think that the reason for the low compatibility of Kinect with manual measurement may be due to the standard difference in the manual measurement method of the anatomical land markers used in the measurement of Shoulder width.

## Discussion

In our research, in which we compared the measurements performed with the Kinect V2 sensor of some anthropometric values of female weightlifting athletes in the under 15 age category with the measurements performed with traditional methods, the high accuracy of the Kinect V2 sensor is remarkable. The traditional measurement method used in this study has not been accepted as the gold standard. Of course, both traditional and Kinect V2 measurement methods have certain limitations. For example, in the traditional measurement method, user-related factors come to the fore. In the Kinect V2 measurement method, environmental and calibration-related factors are important. These factors should not be ignored when comparing the outputs of the methods. The measurements performed in our research showed that there are some differences and concordances between Kinect and manual methods. From the findings in our study, varying degrees of concordance and coherency were observed between Kinect and manual methods, and it was determined that Kinect provided less variability and higher coherency for humerus length (6.34% CV, 4.57 CR), forearm length (5.88% CV, 3.74 CR), trochanter-tibiale laterale length (7.34% CV, 6.46 CR), tibial length (5.35% CV, 4.99 CR), and shoulder width (5.49% CV, 4.55 CR) measurements. In hand length measurements, manual methods (5.84% CV, 2.66 CR) provided slightly more coherence results. These findings indicate that Kinect may be a suitable tool for some measurements (humerus length, forearm length, trochanter-tibiale laterale length, tibial length, shoulder width), but manual methods are preferable for hand length measurements. These results suggest that Kinect V2 technology can be an effective tool in athlete performance assessments. This research specifically focuses on adolescent female weightlifters, a group selected due to their regular training schedules and high level of consistency, as well as their international competitive experience. Moreover, this research compared data obtained from athletic female weightlifters, unlike previous researches conducted on the general population. Also, future research should explore the applicability of Kinect V2 in diverse athletic populations to clarify its broader potential and limitations.

In recent years, the Kinect V2 depth sensor has become an important tool for human body measurements that do not require physical contact. Research demonstrates the usability and accuracy of this sensor in different areas. Adikari’s study [22] showed that Kinect V2 provided high concordance in height and length measurements with an error rate of less than 5%. However, higher error rates were observed in chest, stomach, and waist circumference measurements, and recommendations were made for sensor positioning to increase accuracy in these measurements. In this regard, Krzeszowski et al. highlighted the ability of Kinect V2 to create a 3D human model with depth data and reported measuring various anthropometric parameters (body height, arm length, hip circumference, etc.) with satisfactory accuracy using this model [31]. The study reveals that Kinect offers results concordant with traditional methods, but accuracy needs to be improved in some measurements. Similarly, Park’s research [28] highlights the Kinect V2’s low-cost, portable, and rapid measurement capabilities. In the research, a large number of anthropometric measurements were performed with low error rates, and it was stated that the system is an effective option in cases where a large number of measurements are required in a short time. The applicability of such methods in a wide range of areas from health care to fashion has been emphasized. Other studies have also reported that Kinect is suitable for tracking certain body points and that the repeatability of the measurements is at an acceptable level, but it has been stated that external factors in the measurement process should be controlled and sensor tracking should be improved [53]. The advantages provided by Kinect make it a usable tool in different areas. Findings from our research indicate that the Kinect sensor offers less variability and higher coherency than manual methods in various anthropometric measurements of U-15 weightlifters. Kinect provided reliable results in measuring humerus length, forearm length, trochanter-tibiale laterale length, tibial length, and shoulder width. However, the correlation between the measurement methods showed different levels of reliability. The concordant and low variability provided by Kinect make it a useful tool in different fields, especially where fast and non-contact measurements are important.

A study on estimating human body segment lengths using Microsoft Kinect V2 and X-ray absorptiometry reported a strong correlation between the two methods for arm and forearm length measurements in children, demonstrating the reliability of Kinect-based measurements (left arm: *R* = 0.982, ICC = 0.970; right arm: *R* = 0.972, ICC = 0.961 forearm length; left forearm: *R* = 0.964, ICC = 0.937; right forearm: *R* = 0.944, ICC = 0.938). The authors reported that the high concordance and correlation values observed indicated that Kinect could be used as a reliable anthropometric measurement tool for children [54]. In another study where arm length measurements were performed using the Kinect V2 depth sensor, arm length was calculated by taking the average of the distance from the right and left shoulders to the wrist. In this study, it was reported that the measurements obtained from the Kinect sensor showed good concordance with an error rate of 4.80% when compared to manual measurements. It has been stated that the data obtained show that the sensor offers high accuracy in arm length measurements and has the capacity to make measurements without requiring physical contact. The authors stated that these results support Kinect’s ability to make accurate measurements without requiring contact and its potential in different application areas [22]. Although the ICC values of the humerus length and forearm length measurements obtained in our study showed low reliability (ICC 0.372); the results obtained revealed that Kinect measurements exhibited less variability (CV 5.88% vs. 8.51%) and more coherency (CR 3.74 vs. 5.54%) than manual measurements, and it was observed that Kinect measurements showed less variability and more coherency than manual measurements. The study examined the feasibility of static anthropometric measurements using the Kinect sensor and discussed how various anthropometric measurements such as lower extremity, upper extremity, and shoulder width can be determined through Kinect. In light of the data obtained from the study, the authors explained that Kinect technology has the potential to provide faster and more efficient anthropometric data compared to manual measurements. These results show that the Kinect sensor is a suitable measurement tool, especially for static situations, and it is also emphasized that Kinect is highly dependent on the person’s position and immobility when performing these measurements, and this may affect the accuracy of the measurement results [54, 55] reported that there was a very strong correlation between the measurements of the lower extremity segments in the group of children included in their study. The authors stated that the data obtained showed good concordance in general between Kinect and X-ray absorptiometry methods in lower extremity length measurements and that Kinect could be used as a potential tool in ergonomic evaluations. Studies in the literature show that Kinect can be used as a suitable measurement tool in lower extremity anthropometric assessments. The data obtained in our research also reveal that Kinect can provide coherence results between measurements performed with manual methods and this supports the literature findings. It is observed that the data of young age group children included in Wang’s [54] research are concordant to a certain extent with the data of trochanter tibiale laterale and tibial length of the lower extremities of U-15 athletes included in our research. On the other hand, in our research, it was determined that the upper leg data we measured from two different points with traditional methods differed significantly from the data in both our own research and Wang’s research [54].

In the study using the Kinect V2 depth sensor, shoulder width was measured in 3D without physical contact by calculating the distance between the right and left shoulder skeletal points. Research results show that the Kinect sensor offers acceptable accuracy with an error rate of 3.60% when compared to manual measurements. The authors stated that these data demonstrate Kinect’s ability to make accurate measurements without physical contact and its potential in various application areas [22]. In a study analyzing Kinect-based anthropometric measurements for ergonomic designs compared to traditional methods, it has been reported that the Kinect sensor is suitable for making anthropometric measurements and can effectively measure body points such as head, shoulder, elbow, and knee when positioned correctly to the sensor. Comparisons with control group data and other anthropometric data obtained with traditional methods showed that Kinect data are reliable. It has been stated that Kinect is presented as an effective alternative to traditional measurement tools, especially in dynamic environments and applications requiring rapid data collection. The findings have been explained as increasing the potential of Kinect technology as an anthropometric measurement tool and expanding its application possibilities in areas such as ergonomic product design and workplace arrangements [53]. In our research, it was determined that although ICC showed moderate reliability in shoulder width measurements (ICC 0.477), Kinect measurements exhibited less variability (CV 5.49% vs. 7.43%) and be more coherence (CR 4.55 vs. 6.62%) than manual measurements.

Jamil’s research [55], in which anthropometric measurements (hand length) performed using the Kinect sensor were compared with data obtained with traditional methods, the error in the data obtained with Kinect was measured as 2.16%. They stated that the Kinect sensor can provide sufficiently accurate results in perceiving the dimensions of the human hand, but small errors may occur in areas that require dynamic and detailed information, such as the hand. In another study, it was stated that the accuracy of hand measurements performed with the Kinect V2 sensor was quite high. The research used 3D point cloud data from Kinect V2 to accurately measure various geometric parameters of the hand (volume, area, length, width, finger dimensions, etc.), and these measurements were found to be in good concordance with general size measurements reported in the literature. It was reported that the accuracy of the measurements was achieved with a precision level better than 1 mm through the high resolution of the Microsoft Kinect V2 sensor, and that calibration processes were applied to increase the accuracy of hand measurements, and linear regression models were used to achieve this accuracy [56]. In our research, our comparisons of hand length measurements showed that there was a significant difference between Kinect and manual measurements, and the low ICC value showed that manual measurements were reliable in terms of accuracy and coherency (CV 5.84% etc. 8.83%; CR 2.66 etc. 3.6%). It has been suggested that the differences between the two methods in hand length measurements may be due to the distance of the Kinect device to the user, the light conditions of the environment, and especially the difficulties of the device in fully detecting the extremities of the lower extremities, such as the hand. The limitations of our study are related to the validation of the method; the proposed method was tested only for some selected upper-lower extremity and shoulder width anthropometric parameters. Another limitation relates to our research group. Parameter estimation was conducted only on Turkish Weightlifting National Team U-15 female weightlifting athletes. The method has not been tested on males or other age groups (youth age group, young people, and adults) of weightlifters. However, since the entire universe of elite U-15 female weightlifting athletes constituted our sample group, the number of athletes was limited to 12. In future studies, the number of sample groups can be increased by including U-15 female weightlifters participating in national weightlifting championships.

## Limitations

The limitations of this study include the validation of the method for only selected anthropometric parameters (certain upper-lower extremities and shoulder width). Another limitation was related to the sample group, which was limited to 12 elite U-15 female weightlifters from the Turkish Weightlifting National Team. Therefore, the findings may not be generalizable to other age groups, genders, or larger populations. Future studies should include broader groups and larger samples to enhance generalizability.

## Conclusions

Certain anthropometric parameters of U-15 adolescent female weightlifting athletes were measured and tested, using the Kinect V2 depth camera. Experimental studies have shown that the proposed system provides satisfactory accuracy for many parameters. These results support the usability of the method in anthropometric measurements. We believe that using Kinect could offer a different approach for future anthropometry studies with athletes, through its features such as more advanced depth perception and high-resolution imaging capacity.

## Practical applications

This research may offer significant potential for the applicability of Kinect sensors, especially in sports fields that require non-invasive and real-time measurements. In order to overcome the limitations of the current technology and enable wider use, continuous improvements as well as optimization of some anthropometric measurement protocols and algorithm sensitivities are required. The development of customized solutions could enable such technological imaging methods to find wider use in sports activities, providing significant benefits to athletes, coaches and sports scientists.

## Data Availability

Dataset available on request from the correspondence. The raw data supporting the conclusions of this article will be made available by the authors on request.
